# Esketamine new tool for resistant depressive disorder. About a case

**DOI:** 10.1192/j.eurpsy.2024.1108

**Published:** 2024-08-27

**Authors:** M. Valverde Barea, C. Mata Castro, P. Vargas Melero, A. España Osuna

**Affiliations:** Psychiatry, H.U. Jaén, Jaén, Spain

## Abstract

**Introduction:**

Depressive disorders represent the main cause of disability in the world, due to its prevalence, its impact on the patient’s quality of life and its role as one of the main risk factors for suicide. Current antidepressant treatments can take weeks to take effect and months to achieve response and remission.It is estimated that up to 30% of patients with major depressive disorder (MDD) are resistant to antidepressant treatment, in addition, approximately 30-45% of patients with depression do not achieve an adequate response to the first antidepressant treatment.According to the STAR*D study, the more lines of treatment are required, the lower remission rates are estimated, as well as higher relapse rates during the follow-up phase.With the appearance of intranasal dosage esketamine allows the release directly to the central nervous system, the mechanism of action of esketamine is based on the antagonism of the NMDA receptor, which entails the modulation of the excitatory transmission of glutamate and the release of BDNF,activating neurotrophic signaling and synaptogenesis.

**Objectives:**

The objective is to expose the response after treatment with intranasal esketamine in a case of resistant depression.

**Methods:**

A 55-year-old female patient, diagnosed with resistant recurrent depressive disorder.The patient had undergone treatment with different therapeutic lines with antidepressants, and potentiations with antipsychotics, observing little response in the current episode, for which reason we evaluated the indication of intranasal Esketamine. Scales: MADRS (Montgomery Asberg Depression rating scale) =37, Hamilton Depression Scale=25, PHQ-9=20, indicating severe depression.

**Results:**

After starting treatment with intranasal esketamine, an early response was observed. After the first month of treatment, mild depression was scored at MADRS=10 and moderate depression at Hamilton=14, PHQ-9=12, and at week 14 of treatment, it was scored mild depression in both MADRS and Hamilton. Intranasal 56mg esketamine plus 20mg escitalopram, 30mg mirtazapine and 5mg aripiprazole.

**Conclusions:**

Intranasal esketamine offers a rapid reduction in depressive symptoms maintained over time, reducing the risk of relapse and with a favorable tolerability profile, so its use in depression resistant to treatment presents a great advance.

**Disclosure of Interest:**

None Declared

